# Utility of modified Faine’s criteria in diagnosis of leptospirosis

**DOI:** 10.1186/s12879-016-1791-9

**Published:** 2016-08-24

**Authors:** Kanchana Bandara, Manjula Manoji Weerasekera, Chinthika Gunasekara, Nilantha Ranasinghe, Chamil Marasinghe, Neluka Fernando

**Affiliations:** 1 General Sir John Kotelawala Defense University, Rathmalana, Sri Lanka; 2 Department of Microbiology, Faculty of Medical Sciences, University of Sri Jayewardenepura, Gangodawila, Tangalle, Sri Lanka; 3 Base hospital Tangalle, Colombo, Sri Lanka; 4 Department of Medicine, Faculty of Medical Sciences, University of Sri Jayewardenepura, Gangodawila, Nugegoda, Sri Lanka

**Keywords:** Leptospirosis, Modified Faine’s criteria, Immunochromatography

## Abstract

**Background:**

Leptospirosis is a globally emerging zoonotic disease and an important public health threat in developing countries. Diagnosis of leptospirosis is mainly based on clinical presentations in resource poor countries. World Health Organization (WHO) has introduced “Faine’s criteria” for diagnosis of leptospirosis. This study was conducted to evaluate the usefulness of modified Faine’s criteria (with amendment) 2012 to detect leptospirosis in resource poor settings.

**Methods:**

Blood samples of 168 patients who fulfilled the inclusion criteria admitted between January 2013 to January 2014 were tested by a commercial immunochromatographic assay (Leptocheck WB, India), microscopic agglutination test (MAT) and polymerase chain reaction (PCR) methods. Leptospirosis was confirmed by a single MAT titre ≥1:400 and / or by a positive PCR. Diagnosis of leptospirosis was made using the clinical, epidemiological and laboratory data according to modified Faine’s criteria (with amendment) 2012.

**Results:**

Leptospirosis was confirmed in 39 % (*n =* 66) by MAT and/or PCR. When modified Faine’s criteria (MAT ≥ 1.400 &/ or PCR), was evaluated against LERG confirmed cases sensitivity, specificity, positive predictive value and negative predictive values were 95.45 %, 56.86 %, 58.88 %, 95.08 % respectively. The modified Faine’s criteria with rapid immunochromatographic assay only had a sensitivity, specificity, positive predictive value and negative predictive value 89.39 %, 58.82 %, 58.42 %, and 89.55 % respectively.

**Conclusion:**

The modified Faine’s criteria which utilized only immunochromatographic assay (leptocheck IgM) in Part C was found to be useful tool for diagnosing leptospirosis in a resource poor setting.

## Background

Leptospirosis is a globally emerging zoonotic disease, and an estimated one million severe cases occur each year, with a case fatality up to 40 % [1]. Recent epidemiological data show a rising trend in leptospirosis from all over Asia Pacific region with increased hospital admissions, posing a huge public health threat [2]. In Sri Lanka the annual disease incidence is reported to vary between 31 to 164 per 100, 000 population [3] with high mortality rate among severe cases [4]. Cattle have also been recognized as important reservoirs in addition to rats [5]. This can be mainly attributed to the expansion and distribution of domestic cattle and livestock industry thus posing an increased risk of community exposure to infected animals.

Clinical manifestation of leptospirosis mimics common life threatening infections such as dengue and hanta viral infections [6, 7]. Although prompt treatment with antibiotics can reduce the severity of leptospirosis, diagnosis is often delayed resulting in high mortality. Leptospirosis can result in severe complications such as renal failure, meningitis, pulmonary haemorrhage and multi organ failure [8]. Increasing incidence of the disease in resource poor countries like Sri Lanka highlights the need for more precise and discriminative criteria for diagnosis of leptospirosis. Although specific diagnostic tools such as culture, serology and molecular methods are available, clinical diagnosis stands as the mainstay of disease diagnosis in resource poor countries.

World Health Organization (WHO) has introduced “Faine’s criteria” for diagnosis of leptospirosis, based on clinical history (Part A) and epidemiological history (Part B) supported by laboratory parameters (Part C) [9]. Faine’s criteria has since been amended to increase the sensitivity of diagnosis (Table 1) [10]. The main objective of the present study is to evaluate the usefulness of modified Faine’s criteria (with amendment) 2012 as a diagnostic method to diagnose leptospirosis in Sri Lanka.

**Table 1:** System of scoring using the Modified Faine’s Criteria (with amendment) 2012 for the diagnosis of leptospirosis [10]

Part A: Clinical data	Score
Headache	2
Fever	2
Fever >39 °C	2
Conjunctival suffusion	4
Meningism	4
Myalgia	4
Conjuctival suffusion + Meningism + Myalgia	10
Jaundice	1
Albuminuria / Nitrogen retention	2
Haemoptysis/ dyspnoea	2
Part B: Epidemiological factors	Score
Rainfall	5
Contact with contaminated environment	4
Animal contact	1
Part C: Bacteriological and Laboratory Findings	Score
*Isolation of leptospira in culture – Diagnosis certain*	
PCR^a^	25
Positive serology	
ELISA^b^ IgM positive^f^	15
SAT^c^ positive^f^	15
Other rapid tests^df^	15
MAT^e^ – single positive in high titer^f^	15
MAT^e^ – Rising titer / seroconversion (paired sera)	25
Presumptive diagnosis of leptospirosis is made of:	
Part A or Part A & Part B score : 26 or more	
Part A, B, C (Total) : 25 or more	
A score between 20 and 25 suggests leptospirosis as a possible diagnosis.	

^a^ Polymerase Chain Reaction

^b^ Enzyme Linked Immunosorbant Assay

^c^ Slide Agglutination Test

^d^ Other rapid tests– Latex agglutination test / Leptodipstick / LeptoTek lateral flow / Lepto Tek Dri Dot test

^e^ Microscopic Agglutination Test

^f^ Any one of the tests only should be scored

## Methods

The study was carried out at a Tertiary care hospital which caters for around 2000 inpatients and selected secondary care hospitals in Western and Southern provinces in Sri Lanka. All patients clinically suspected of leptospirosis (*n =* 203), admitted to the medical wards between January 2013 to January 2014 were screened based on the inclusion criteria.

The inclusion criteria of our study was based on “suspected case definition” given in Communicable Disease Epidemiology Profile Sri Lanka, World Health Organization [11]. It included presence of acute febrile illness with headache, myalgia and prostration associated with any other clinical signs including conjunctival suffusion, anuria, proteinuria, or oliguria, jaundice, cough, haemoptysis and breathlessness, haemorrhages (in intestines and lung), meningeal irritation, cardiac arrhythmia or failure, skin rash and exposure to contaminated environment. Contaminated environment includes exposure to water possibly contaminated with leptospira (paddy fields/ agricultural fields, domestic sewage, live stock waste, flood water, construction site, river, canal, ditch, recreational activities like rowing, white water rafting etc.), exposure to animals (rodents, livestock, domesticated and wild), and occupational exposure (farmers, outdoor labourers, fisherman, abattoir worker, veterinarians, military etc.) within a period of one month.

Ethical approval for the study was granted from Ethical review Committee of University of Sri Jayewardenepura, Sri Lanka (Application No.702/12).

### Case definition of leptospirosis

A definitive case was classified based on the WHO LERG report, by symptoms consistent with leptospirosis and a single MAT titre ≥1:400 or/and by detection of *Leptospira* DNA by PCR. A presumptive case was identified as symptoms consistent with leptospirosis and presence of IgM antibodies [12].

Diagnosis of leptospirosis was made according to the modified Faine’s criteria (with amendment) 2012 using clinical data (Part A), epidemiological data (Part B) and laboratory data (Part C) [10]. Faine’s score was obtained for each patient using clinical, epidemiological and laboratory data according to Table 1. A score of 25 or more for parts A + B + C or a score of 26 or more for parts A and B was considered as presumptive leptospirosis (Table 1).

Data on socio demographic characteristics and environmental exposure were gathered using a pretested interviewer administered questionnaire. Results of the basic laboratory tests (full blood count (FBC), urine analysis, renal function test, liver profile etc.), clinical presentation and outcome of the patient were gathered using the bed head tickets. Blood samples were collected from patients after obtaining informed written consent. Whole blood (3 ml) was collected into a plain tube to obtain serum for rapid immunochromatographic assay and MAT. Additional 2 ml of whole blood was collected into an EDTA tube for DNA extraction. All samples were transported on ice to the Department of Microbiology, University of Sri Jayewardenepura within 24 h for processing.

### IgM immunochromatographic assay and microscopic agglutination test (MAT)

Leptospira infection was presumptively diagnosed by detecting leptospira specific IgM using a rapid immunochromatographic assay kit (Leptocheck WB, Zephyr Biomedicals, India) following the manufacturer’s instructions. Microscopic agglutination test was done in order to obtain single MAT antibody titers using the genus specific *Leptospira biflexa* serovar Patoc (1) strain and a single titre of ≥1:400 was considered as positive according to LERG criteria [12].

### PCR amplification

Leptospira DNA was extracted from 200 μl of EDTA blood samples by using QIAamp DNA blood mini kit (QIAGEN GmbH, Hilden, Germany) according to the manufacturer’s instructions. Eluted DNA was quantified and purity was checked using Nanodrop 2000/2000C spectrophotometer (Thermo fisher scientific, USA).

The extracted DNA was used to amplify 16S ribosomal DNA gene of pathogenic and intermediate *Leptospira* species by nested PCR. A single tube nested PCR was carried out using rrs-outer F, rrs-outer-R, rrs-inner-F, rrs-inner-R [13] primers adhering to the method described previously [14]. Nested PCR resulted in a 547 bp product.

### Data analysis

Data were analysed using Statistical Package for Social Science (SPSS version 15). Sensitivity, specificity, positive and negative predictive values were calculated at 0.05 significance.

## Results

**Fig. 1:**
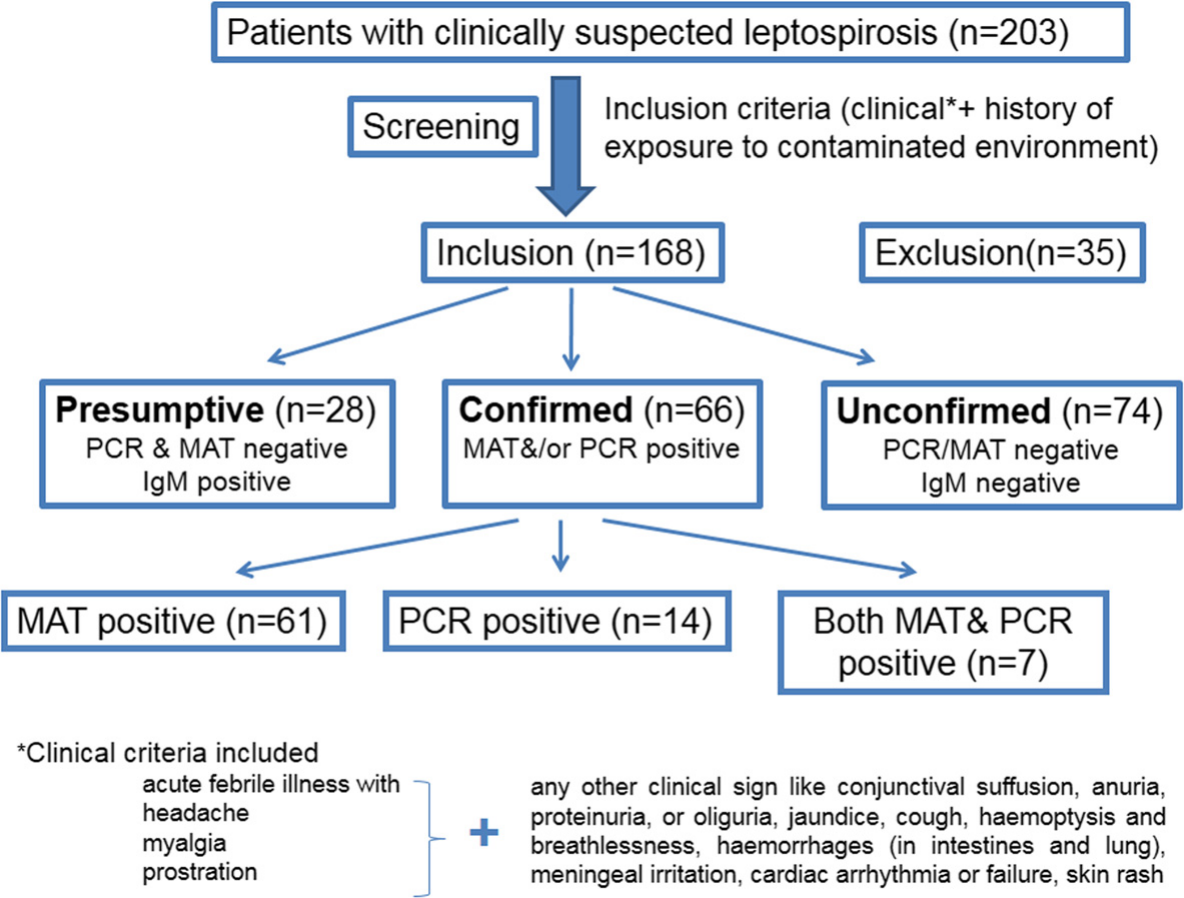
Diagnostic and laboratory characteristics of clinically suspected leptospirosis

Two hundred and three patients having acute febrile illness were recruited and 168 patients had clinically suspected leptospirosis according to the inclusion criteria (Fig. 1). Out of these patients 66 had confirmed leptospirosis based on LERG case definition, having a positive PCR test (*n =* 14), MAT titre ≥1:400 (*n =* 61) or both (*n =* 7). Among the 61 MAT titre ≥1:400 patients, 55 (90 %) had a titre of ≥1:800. There were 28 presumptive cases identified only by the presence of IgM (LERG case definition). Out of the 168 clinically suspected cases, 84 patients (50 %) were leptospira IgM positive.

Out of the 168 clinically suspected patients 43 % (*n =* 72) were farmers, 34.5 % (*n =* 58) were laborers. When the confirmed leptospirosis cases were analysed, 45 % (30/66) were farmers while 40.9 % (27/66) were labourers. Among the 102 unconfirmed cases, 42 patients (41 %) were farmers while 31 (30 %) were labourers. There was no significant difference in the occupation between confirmed and unconfirmed cases.

Among the 66 leptospirosis confirmed patients 75.8 % (*n =* 50) had exposure to contaminated environment and 40.9 % (*n =* 27) had exposure to animals including cattle, pigs, dogs and rats. All 168 cases had some form of exposure to contaminated environment as samples were collected during the outbreaks associated with the monsoon rains.

Most common symptoms among confirmed cases of leptospirosis in this study were fever, myalgia, headache, and albuminuria/nitrogen retention. Among the features, icterus (*p =* 0.000) and albuminuria/ nitrogen retention (*p =* 0.020) showed significant association with leptospirosis (Table 2). On admission to the hospital median duration of fever was 6 days (±2.5). When the results of PCR were considered, 12 out of 14 (85.7 %) patients were positive within the first 6 days of fever. While only 2 out of 14 (14.2 %) were positive later. When a MAT titre of ≥ 1:400 was considered 22 (36 %) out of 61 were positive within the first 6 days of fever while 39 (63.9 %) were positive after 6 days.

**Table 2:** Comparison of clinical features among clinically suspected leptospirosis patients; with confirmed and without a confirmed diagnosis based on MAT and /or PCR

Clinical features	Percentage of patients with confirmed diagnosis of leptospirosis^a^ (*n =* 66)	Percentage of patients without a confirmed diagnosis of leptospirosis^b^ (*n =* 102)	p value
Fever	100	100	-
Headache	72.7	73.5	0.909
Myalgia	81.8	85.3	0.549
Muscle tenderness	45.5	35.3	0.118
Vomiting	54.5	44.1	0.186
Conjunctivitis	19.7	11.8	0.158
Icterus	33.3	8.8	0.000*
Conjunctival haemorrhage	25.8	14.7	0.075
Meningitis	15.2	7.8	0.135
Dyspnoea	15.2	8.8	0.206
Albuminuria/nitrogen retention	68.2	50	0.020**

^a^ Confirmed diagnosis of leptospirosis included patients having symptoms consistent with leptospirosis and a single MAT titre ≥ 1:400; and / or Leptospira DNA detected by a method based on the polymerase chain reaction (PCR)

^b^ Patients negative for MAT and / or PCR (This includes presumptive and unconfirmed cases)

* *p <*0.001, **p < 0.05

When the 168 patients were evaluated by the modified Faine’s criteria (2012) using part A (clinical data) and B (epidemiological factors) only 11 patients satisfied the presumptive identity of leptospirosis. Referring to parts A, B and C (bacteriological and laboratory findings) 95 patients were presumptively identified as having leptospirosis. The performance characters obtained when considering only part A + B, and parts A + B + C (i) MAT ≥ 1:400 &/ or PCR, (ii) MAT ≥ 1:800 &/ or PCR, (iii) rapid immunochromatographic test (Leptocheck WB, Zephyr Biomedicals, India only) are described in Table 3.

**Table 3:** Performance characters of Modified Faine’s Criteria (with amendment) 2012

Performance character	A + B only	A + B + C MAT ≥1.400 &/ or PCR	A + B + C MAT ≥1.800 &/ or PCR	A + B + C rapid immunochromatographic test^a^ only
Sensitivity (%)	39.39	95.45	98.31	89.39
Specificity (%)	79.41	56.86	55.05	58.82
(+) Likelihood Ratio	1.91	2.21	2.19	2.17
(−) Likelihood Ratio	0.76	0.08	0.03	0.18
PPV (%)	55.32	58.88	54.21	58.42
NPV (%)	66.94	95.08	98.36	89.55

A: Clinical history, B: Epidemiological history, C: Laboratory parameters

^a^(Leptocheck WB, Zephyr Biomedicals, India)

Modified Faine’s criteria was evaluated using LERG confirmed leptospirosis cases as the bench mark. When the modified Faine’s criteria (2012) was evaluated for parts A + B only, sensitivity of 39.39 % (26 Faine’s A + B positive /66 confirmed by LERG criteria) and a specificity of 79.41 % (81 Faine’s A + B negative /102 unconfirmed by LERG criteria) was detected (Table 3). When considering parts A + B + C (MAT ≥ 1:400 & / or PCR), results showed a sensitivity of 95.45 % (63 Faine’s A + B+ C positive /66 confirmed by LERG criteria). The specificity of this test was 56.86 % (58 Faine’s A + B + C negative /102 unconfirmed by LERG criteria).

However when parts A + B + C (MAT ≥ 1:800 & / or PCR was considered, the test showed the highest sensitivity of 98.31 % (58 Faine’s A + B+ C positive /59 confirmed by LERG criteria with a MAT titre ≥ 1:800) and a specificity of 55 % (60 Faine’s A + B+ C negative /109 unconfirmed by LERG criteria with a MAT titre ≥ 1:800). On evaluation of the modified Faine’s criteria for parts A + B + C and immunochromatographic assay only, a sensitivity of 89.39 % (59 Faine’s A + B+ C positive with rapid test only /66 confirmed by LERG criteria with a MAT titre ≥ 1:400) and a specificity of 58.82 % (60 Faine’s A + B+ C negative /102 unconfirmed by LERG criteria with a MAT titre ≥ 1:400) was detected. The highest negative predictive value was seen when parts A + B + C (MAT ≥ 1:800 & / or PCR) was considered (98.36 %). The highest positive predictive value was obtained for Faine’s A + B + C MAT ≥ 1.400 &/ or PCR (58.88 %) (Table 3).

## Discussion

The present study evaluated the utility of modified Faine’s criteria (with amendment) 2012 for the diagnosis of leptospirosis in Sri Lanka. Diagnosis of leptospirosis in the rural hospitals with minimal facilities is a major challenge. This study aimed at facilitating diagnosis using a simple method utilizing clinical, epidemiological and basic laboratory investigations available in rural hospitals. Leptospirosis mimics a number of other important infections due to the nonspecific nature of the clinical presentation and therefore can be misdiagnosed frequently. Further, confirmatory laboratory tests for leptospira such as MAT and PCR are only available in reference laboratories where the facilities for leptospira culture and molecular biology are available. Therefore, it is essential that feasible and reliable diagnostic criteria based on clinical symptoms and basic laboratory tests be identified and their diagnostic utility evaluated for resource poor settings such as Sri Lanka.

The “modified Faine’s criteria (with Amendment) 2012” is user friendly and sensitive where in Part A: haemoptysis/ dyspnoea, Part B: rainfall and part C: rapid immunochromatographic assay has been included [10]. Sri Lanka being a humid, tropical country hyper endemic for leptospirosis, where the rural economy of the country mainly relies on paddy cultivation and small holder dairy industry, exposure to water buffaloes and cattle is very common during farming. Further rapid urbanization has increased rodent populations and further increased human contact with wild and domesticated animals. Most importantly many areas of the country are prone to flood during the two monsoon seasons which arrived during the period of the study.

The tool used to diagnose the disease in its early course should be sensitive and should have a high PPV to be able to give an accurate diagnosis and thus deliver prompt and specific treatment for a favorable clinical outcome.

According to the current study utility of combination of clinical (part A), epidemiological (part B) and laboratory criteria (part C) is higher than clinical and epidemiological criteria only. In the study, among the several diagnostic criteria used sensitivity is higher with MAT and / or PCR. But due to the limited availability of these tests alternative test that are easy to use are needed. In this scenario, when rapid immunochromatographic test (Leptocheck WB, Zephyr Biomedicals, India) is combined with clinical and epidemiological criteria, it will help in diagnosis of leptospirosis as it has a good sensitivity (89.39 %) although the specificity is slightly lower (58.82 %) compared to MAT and / or PCR.

A study done by Brato, et al., validated the Faine’s criteria (Part A + B) in the diagnosis of leptospirosis using MAT as gold standard [15] in 53 adult patients. He reported a sensitivity, specificity and positive predictive value of 33 %, 65 %, and 67 % respectively. In the present study, using the modified Faine’s criteria, we report a high sensitivity of 95.45 % using MAT and or PCR as confirmatory tests. The inclusion of MAT and/ or PCR for leptospira diagnosis may be an important factor contributing to higher sensitivity when A + B + C are considered. However in this study, we further evaluated A + B and a simple immunochromatographic assay (Leptocheck WB, Zephyr Biomedicals, India). This immunochromatographic assay is simple to carry out, rapid bedside test and do not need sophisticated equipment hence is important in leptospira diagnosis in resource poor settings. Further, the PPV (58.42 %) observed in the current study using only immunochromatographic assay (Leptocheck WB, Zephyr Biomedicals, India) supports the above finding. Although there are other studies done to evaluate utility of Faine’s criteria with comparatively higher PPV, they have not appreciated the use of immunochromatographic assay only.

Another study done by Bhatia et al. has shown poor positive predictive value for IgM Leptocheck, Macroscopic slide agglutination test, enzyme linked immunosorbant assay (ELISA) and modified Faine’s criteria (14.3 %, 6.5 %, 8.7 % and 21 %) taking MAT as the gold standard [16]. However, PPV of current study is far better than that reported by Bhatia. The positive predictive values described in various studies are influenced by several factors, including the study design, sample size, gold standard used and the prevalence of the disease in the country. In a region with high prevalence of disease more true positives will be identified resulting in a higher PPV, compared to a region with low disease prevalence. Therefore, results of these studies cannot be directly compared with the current study. Furthermore the PPV of modified Faine’s criteria A + B + C ≥ 1:400 MAT &/ or PCR, and the PPV of modified Faine’s criteria A + B + C and rapid immunochromatographic assay were comparable in the present study which suggests that the rapid immunochromatographic assay to be a cost effective, rapid and useful test when used in combination with the modified Faine’s criteria. This is indeed a positive finding for the clinicians working in rural underprivileged settings where diagnosis has to be made with limited resources.

The basis for calculation of the performance parameters in this study was based on the WHO LERG criteria as the reference which considered a positive PCR and or a single titre MAT ≥1:400. While PCR is a highly sensitive molecular detection method for diagnosing leptospirosis, its performance is affected by factors such as late presentation to the hospital for investigations, prior antibiotic treatment and the type of PCR which could lead to a false negative result. In this study although a nested PCR was used to improve the sensitivity it was found to be less (100 cells /ml blood) compared to a real time PCR, which would have contributed to false negative results. Further a limitation in the present study was the use of a single titre MAT for confirmation. In the current resource poor settings in the country although achieving paired sera is important due to the early discharge of patients and the lack of a follow up facilities; collection of paired sera could not be done. In the present study most patients presented on 6th day of fever, while it is known that MAT antibody titres peak in the 2nd to 3rd week of infection. Single MAT titre of ≥1:400 although given in the LERG criteria as confirmatory diagnostic criteria, would have contributed to false positive results. In addition, Sri Lanka being a hyper endemic region for leptospirosis, antibodies from past infection is known to persist for a long time. These factors could result in misclassification of true infected and non infected in this study which was conducted in a resource poor setting and are limiting factors. It is important to highlight that one of the most influential factors in determination of the predictive value of the test is the disease prevalence. Our study was limited to several selected hospitals in two provinces in Sri Lanka, which does not represent the entire population of the country. Further test accuracy and the study design also influence the predictive value. Therefore these factors were limitations when comparison was done with studies published in other countries.

## Conclusion

The present study evaluated the utility of modified Faine’s criteria (with amendment) 2012 for leptospirosis diagnosis, and found it to be highly sensitive and specific. Further for the first time globally, modified Faine’s criteria which utilized only immunochromatographic assay (Leptocheck WB, India) in Part C was found to be useful in arriving at a presumptive diagnosis of leptospirosis. This finding makes it a useful tool for diagnosing leptospirosis in a resource poor setting.

## Abbreviations

ELISA: Enzyme linked immunosorbant assay
MAT: Microscopic agglutination test
PCR: Polymerase chain reaction
PPV: Positive predictive value
